# The Interplay between Gender and Duration of Hospitalization Modulates Psychiatric Symptom Severity in Subjects with Long COVID-19

**DOI:** 10.3390/brainsci14080744

**Published:** 2024-07-25

**Authors:** Alessio Simonetti, Antonio Restaino, Claudia Calderoni, Emanuela De Chiara, Antonio Maria D’Onofrio, Salvatore Lioniello, Giovanni Camardese, Delfina Janiri, Matteo Tosato, Francesco Landi, Gabriele Sani

**Affiliations:** 1 Department of Neuroscience, Head-Neck and Chest, Section of Psychiatry, Fondazione Policlinico Universitario Agostino Gemelli IRCCS, 00168 Rome, Italy; 2 Menninger Department of Psychiatry and Behavioral Sciences, Baylor College of Medicine, Houston, TX 77030, USA; 3 Department of Neuroscience, Head-Neck and Chest, Section of Psychiatry, Università Cattolica del Sacro Cuore, 00168 Rome, Italydechiaraemanuela@gmail.com (E.D.C.);; 4 Department of Geriatrics, Università Cattolica del Sacro Cuore, 00168 Rome, Italyfrancesco.landi@unicatt.it (F.L.); 5 Department of Geriatrics, Fondazione Policlinico Universitario Agostino Gemelli IRCCS, 00168 Rome, Italy

**Keywords:** COVID-19, long COVID-19 syndrome, depression, anxiety, psychopathology, hospitalization, gender

## Abstract

Long COVID-19 is characterized by ongoing symptoms or prolonged or long-term complications of SARS-CoV-2 contraction which persist beyond 4 weeks from the initial onset of symptoms. Gender and duration of hospitalization (DH) are key risk factors for developing long COVID-19 syndrome, but their impact and interplay need further study. This research involved 996 long COVID-19 patients, and we compared the levels of general psychopathology, depression, agitated depression, anxiety, and medication use between hospitalized and non-hospitalized males and females. In the hospitalized patients, multivariate regressions assessed the impact of gender, DH, and the interaction of these variables. The females had higher levels of long COVID-19 symptoms, psychotropic drug use, depression, anxiety, and general psychopathology than the males. The non-hospitalized females exhibited more severe agitated depression than the non-hospitalized males. In females, DH was more strongly correlated with the number of psychotropic medications used during long COVID-19. A negative correlation was found between DH and severity of agitated depression in the female patients only. These results highlight that the gender-specific relationship between DH and agitated depression severity should be explored further.

## 1. Introduction

Coronavirus Disease 19, also known as COVID-19, is a syndrome caused by contracting SARS-CoV-2. COVID-19 was first reported in Wuhan, China and subsequently spread globally, becoming a pandemic in March 2020 [1,2]. The symptoms brought about by COVID-19 are mainly mild, but in a considerable number of cases, contracting SARS-CoV-2 might lead to severe complications and death. Observations of COVID-19’s course led to the understanding that pulmonary and systemic alteration brought on by COVID-19 might persist for months after the acute infection. The persistence or delayed onset of at least one symptom for at least 4 weeks after COVID-19 which cannot be accounted for by an alternative diagnosis [3] has been defined as long COVID-19. Long COVID-19 symptoms include fatigue, dyspnea, cough, altered gastrointestinal function, musculoskeletal problems, and memory and concentration impairment [3,4]. Psychiatric disorders, such as anxiety, depression, sleep disturbances, and post-traumatic stress disorder, are also frequently present [5]. Several risk factors have been identified as potential factors for the onset and development of long COVID-19. Among them, gender and duration of hospitalization (DH) are the most relevant ones [6]. However, studies mostly focused on their role in the development of long COVID-19 organic symptoms. When research on possible predictors of long COVID-19 narrows to psychiatric symptoms, the roles of gender and DH are less defined and equivocal. The female gender was associated to depression [7,8,9,10], anxiety [7,9,10,11], somatic symptoms [7], and insomnia [11], whereas other studies reported no effect of gender on these symptoms [7,10,12,13] or a greater prevalence in males [14]. The effect of DH on the psychiatric sequelae of COVID-19 has been investigated less. Fernández-de-Las-Peñas [15] reported that DH correlates with greater anxiety and depression during long COVID-19. In contrast, Matalon et al. [16] and Huarcaya-Victoria et al. [7] reported an absence of any effect of DH on psychiatric symptoms. Therefore, there is a need to clarify the effect of gender and, mostly, the effect of DH on the psychiatric sequelae of COVID-19. Additionally, due to the importance of these two factors, there is a need to evaluate their interplay in modulating long COVID-19-associated symptomatology. The present study’s aim is twofold: to investigate the effect of gender and DH on psychiatric symptoms in subjects with long COVID-19 and to evaluate the possible interplay between these two factors between these symptoms. General psychopathology, anxiety, facets of depression, the range of psychiatric medications, and the total score of each psychometric scale were the variables considered. It was expected that a female gender and longer DH would be associated with greater severity of psychiatric symptoms and increased medication intake. Also, it was expected that longer DH in females would correlate with a higher number and severity of psychiatric symptoms compared with males.

## 2. Materials and Methods

### 2.1. Sample

The study was conducted by the Gemelli Against COVID-19 Post-Acute Care Study Group. Enrolled subjects were those who had contracted SARS-CoV-2 and developed long COVID-19. Although there is no consensus on the definition of this syndrome, the most commonly used criteria were adopted. More specifically, we defined long COVID-19 as follows: persistent symptoms or delayed or long-term complications of COVID-19 which last beyond 4 weeks and cannot be accounted for by an alternative diagnosis [3]. Such a diagnosis was made after careful medial anamnesis and after reviewing patients’ clinical charts. In the case of the duration of symptoms not being clear, the subject was excluded from the study.

The inclusion criteria were (1) the faculty to provide written informed consent; (2) an age ranging between 18 and 75 years old; (3) a previous COVID-19 case, as demonstrated by positive COVID-19 tests; and (4) the absence of COVID-19, as demonstrated by a negativite COVID-19 test performed within 48 h from the evaluation. The exclusion criteria were (1) impossibility to reach the evaluation center; (2) severe neurological disorders, such as presence of a recent stroke, intracranial hemorrhage, unstabilized Parkinson’s disease, encephalitis, meningitis, unstabilized epilepsy, multiple sclerosis, or amyotrophic lateral sclerosis; (3) dementia or mild cognitive impairment; (4) unstabilized medical disorders such as tumors; and (5) neurodevelopmental disorders, such as intellectual disabilities, communication disorders, autism spectrum disorders, attention-deficit/hyperactivity disorder, or learning disorders. The enrolled subjects underwent a comprehensive evaluation at the Post-Acute Care Service of Fondazione Policlinico Universitario “Agostino Gemelli” IRCCS of Rome, Italy between 21 April 2020 and 11 July 2022. The patients underwent an extensive assessment process, including a thorough review of their medical history, a comprehensive physical examination, and evaluations by various specialists. Psychiatric evaluations were conducted through a clinical face-to-face interview and through hetero-administered rating scales assessing several psychiatric symptoms, such as general psychopathology, depressive symptoms, anxiety symptoms, and post-traumatic symptoms. The whole evaluation was made with respect to the Italian Ministry of Health recommendations for the pandemic. The study was approved by the Ethical Committee of the Fondazione Policlinico Universitario Agostino Gemelli IRCCS (protocol number: 0013008/20).

### 2.2. Assessment

The data collected included (1) sociodemographic characteristics (e.g., age, gender, employment status, and years of education); (2) medical history before COVID-19; (3) information regarding SARS-CoV-2 infection, such as the time between the onset of COVID-19 and the time of evaluation, symptoms experienced during and after infection, history of hospitalization, and DH; and (4) data from psychiatric evaluations, including personal psychiatric history, the presence or absence of substance abuse prior to COVID-19, rates of consumption of psychotropic drugs before and during COVID-19, the number of general and psychotropic medications prescribed after COVID-19, and the severity of psychiatric symptoms assessed with rating scales. We chose specific rating scales assessing depression, anxiety, and general psychopathology. These scales demonstrated reliability and validity in both clinical and research contexts, ensuring robust and meaningful data collection. Furthermore, the combination of these tools allowed for a thorough evaluation of the most common psychiatric symptoms, which was essential for understanding the multifaceted nature of the conditions under investigation. Details regarding the rating scales used are provided below. The Hamilton Anxiety Rating Scale (HAM-A) [17] was developed to evaluate the severity of anxiety symptoms. With its 14 item structure, the scale assesses both psychological anxiety, such as worries and irritability, and somatic anxiety, which includes physical manifestations. Scores on the HAM-A range from 0 to 56. The Brief Psychiatric Rating Scale (BPRS) [18] acts as a thorough tool for assessing general psychopathology, encompassing anxiety, depression, and psychosis. Using a Likert scale, clinicians can rate the presence and severity of these symptoms from 1 (absent) to 7 (extremely severe). BPRS scores range from 24 to 168, with lower scores indicating less severe psychopathology. The Hamilton Rating Scale for Depression (HAM-D) [17] is employed to assess the severity of depressive symptoms. In this study, the 17 item version of the HAM-D was utilized. Each item measures depressive symptoms experienced over the past week and is rated from 0 (absent) to either 2 or 4 (severe). The Koukopoulos Mixed Depression Rating Scale (KMDRS) [19] has been designed to assess the severity of agitated depression. Scores on the scale range from 0 to 51, with higher scores indicating greater severity of agitated depressive symptoms experienced by the individual. In this work, analyses regarding psychiatric symptoms employed the scales’ total scores instead of cut-offs. This choice was motivated by the unreliability of the severity cut-off. Such unreliability derives from the vast heterogeneity of the cut-off used in clinical studies [20,21,22,23,24] or their unclear relationship with the clinical picture [25].

### 2.3. Statistical Analyses

#### 2.3.1. Sample

Descriptive analyses of the sample were initially performed. The enrolled subjects were then categorized into four subgroups: (1) non-hospitalized males (NHMs); (2) hospitalized males (HMs); (3) non-hospitalized females (NHFs); and (4) hospitalized females (HFs). Between-group differences were analyzed with an χ^2^ test for the nominal variables and multiple one-way analysis of variance (ANOVA) for the continuous variables. In each ANOVA or χ^2^ test, the groups (NHMs, HMs, NHFs, and HFs) were the independent variables, while the sociodemographic characteristics, data regarding SARS-CoV-2 infection and long COVID-19, and data regarding psychiatric evaluation were the dependent variables. Bonferroni correction was applied for multiple comparisons regarding psychometric scales. Tukey tests were used as post hoc tests for continuous variables. With regard to the χ^2^ tests, post hoc testing was performed using Z tests for the independent proportions.

#### 2.3.2. Effect of Gender, DH, and Their Interaction

To investigate the effect of gender, DH, and their interaction on long COVID-19 symptoms and specifically long COVID-19 psychiatric manifestations, multivariate regressions were performed. In each multivariate regression, gender, DH, and their interaction were independent variables. The dependent variables were the number of long COVID-19 symptoms, number of medications during COVID-19, number of psychotropic medications assumed during long COVID-19, and BPRS, HAM-A, HAM-D, and KMDRS total scores. In the case of a significant interaction effect, linear regressions were performed for males and females separately.

#### 2.3.3. Effect of Possible Confounding Variables

The results of the ANOVA and t-tests were corrected for the effect of demographic and clinical variables showing significant differences among groups through multiple ranked one-way analyses of covariance (ANCOVAs). In order to explore the possible effects of other confounding variables, the relationships found between gender and DH, and their interactions with variables related to long COVID-19, linear regressions were performed. In each regression, age, employment, years of education, number of symptoms during COVID-19, psychiatric symptoms during COVID-19, and psychiatric medications during COVID-19 were the independent variables, and the number of long COVID-19 symptoms, number of medications, and number of psychiatric medications assumed during long COVID-19 as well as the BPRS, HAM-A, HAM-D, and KMDRS total scores were the dependent variables. Independent variables showing a significant relationship in the aforementioned analyses entered a multivariate regression along with gender, DH, and their interaction. The number of long COVID-19 symptoms, number of medications assumed during long COVID-19, and number of psychiatric medications taken during long COVID-19 along with the BPRS, HAM-A, HAM-D and KMDRS total scores were the dependent variables.

Statistical analyses were performed using SPSS Statistics 24.0 for Windows (IBMCo., Armonk, NY, USA; 2016).

## 3. Results

### 3.1. Sample

A total of 996 patients were enrolled. The sample had an average age of 52.48 years, with the majority being women (n = 523, 52.51%). Most participants (n = 798, 80.12%) were employed and had an average of 13.81 years of education, while 9.54% of the patients had a positive psychiatric history, and 5.12% had previously received a psychopharmacological prescription before COVID-19. Among the sample, 419 patients (42.03%) had been hospitalized for COVID-19. The results from the ANOVAs and χ^2^-tests are shown in Table 1. Regarding sociodemographic characteristics, the groups exhibited differences in age, employment status, psychiatric history prior to COVID-19, and substance abuse. Post hoc analyses revealed that the NHFs and HFs were significantly older than the HMs. The HMs had higher employment rates than the NHFs and HFs, while the NHFs and NHMs were more frequently employed than the HF. The NHF and HF had positive psychiatric histories more often than the NHMs and HMs. Additionally, the rates of substance abuse were higher among the HMs compared with the NHMs, NHFs, and HFs. Significant differences among groups were observed for the COVID-19-related variables (i.e., the number of symptoms during COVID-19, presence of psychiatric symptoms during COVID-19, and psychopharmacological prescriptions during COVID-19). Post hoc analyses showed that the HFs and NHFs had more symptoms during COVID-19 compared with the HMs and NHMs. The HMs and HFs experienced more psychiatric symptoms during COVID-19 than the NHFs. Furthermore, the HFs and NHFs received psychotropic drugs more frequently during COVID when compared with the NHMs and HMs. Regarding variables related to long COVID-19, the groups differed in terms of the number of long COVID-19 symptoms, total number of medications and psychotropic medications received after COVID-19, and the BPRS, HAM-A, HAM-D, and KMDRS total scores. The number of long COVID-19 symptoms was higher in the HFs and NHFs when compared with the HMs and NHMs. The HFs received more medications after COVID-19 when compared with the NHMs and NHFs. Psychotropic drugs after COVID-19 were more often prescribed for NHFs and HFs relative to NHMs and HMs. Regarding the rating scales, the reliability was good (Chronbach’s α for HAM-D = 0.82; Chronbach’s α for HAM-A = 0.88; Chronbach’s α for BPRS = 0.79; Chronbach’s α for KMDRS = 0.79). The NHF and HF groups showed higher scores on the BPRS, HAM-A, and HAM-D scales when compared with the HM and NHM groups, while the NHFs had higher scores than NHMs on the KMDRS. All the results found survived Bonferroni correction for multiple comparisons.

### 3.2. Effect of Gender, DH, and Their Interaction

Regression analyses revealed a main effect of gender on long COVID-19 symptoms, the number of psychotropic medications during long COVID-19, and the BPRS, HAM-A, and HAM-D total scores. The female gender was associated with a greater number of long COVID-19 symptoms and psychotropic medications during long COVID-19, as well as higher BPRS, HAM-A, and HAM-D total scores. There was also a DH effect related to gender on the KMDRS total scores and the number of medications, as shown in Table 2.

With regard to the interaction effects, linear regressions were conducted separately for males and females. DH was more strongly correlated with the number of drugs and psychotropic drugs taken during long COVID-19 syndrome in females than in males. In females only, there was a negative correlation between DH and the KMDRS scores, whereas in males, no significant relationship emerged. Data are shown in Table 3.

### 3.3. Effect of Possible Confounding Variables

After adjusting for possible confounding variables, the differences in the number of medications prescribed after COVID-19 were no longer significant. The results of the ANCOVAs are present in Supplementary Table S1.

The results from the linear regression analyses are presented in Supplementary Tables S2–S12. Age was positively related with the number of medications taken during long COVID-19. Gender showed a positive relationship with the number of long COVID-19 symptoms, number of psychotropic drugs prescribed during long COVID-19, and the HAM-A, HAM-D, and BPRS total scores. The years of education were negatively associated with the BPRS total scores. The presence of employment was negatively associated with the number of medications and number of psychotropic medications received during long COVID-19, as well as with the BPRS and HAM-D total scores. The presence of a psychiatric history prior to COVID-19 predicted higher BPRS, HAM-A, and HAM-D total scores. The presence of psychiatric medication consumption prior to COVID-19 showed a positive relationship with the number psychotropic medications prescribed after COVID-19 and with the BPRS, HAM-A, and HAM-D total scores. The presence of substance abuse prior to COVID-19 was negatively associated with the number of long COVID-19 symptoms and number of medications prescribed after COVID-19. A longer duration between COVID-19 onset and evaluation in the Gemelli Against COVID-19 Post-Acute Care Center positively predicted the number of long COVID-19 symptoms and number of psychotropic medications received during long COVID-19, while it was negatively associated with the HAM-A total scores. A history of psychiatric symptoms during COVID-19 was positively associated with the number of long COVID-19 symptoms and number of psychotropic medications prescribed during long COVID-19. A higher number of symptoms experienced during COVID-19 was associated with a higher number of long COVID-19 symptoms, as well as higher BPRS, HAM-A, HAM-D, and KMDRS total scores. A greater number of psychiatric medications taken during COVID-19 showed a positive relationship with the number of long COVID-19 symptoms, number of medications taken during long COVID-19, number psychotropic drugs prescribed during long COVID-19, and BPRS score. After adjusting the results concerning the effect of DH and gender interaction for variables demonstrating significant relationships in the linear regression analyses, the relationship between gender, DH, and number of medication prescriptions after COVID-19 remained significant, as well as the relationship between gender, DH, and the KMDRS total score. Additionally, DH and gender interaction and the number of psychotropic medications during long COVID-19 became significant. The results are shown in Supplementary Tables S15–S21.

The latter relationship was then investigated with linear regression run separately for males and females. Females showed a stronger correlation between DH and the number of psychotropic drugs than males. This latter correlation is shown in Table 3.

## 4. Discussion

The results might be summarized as follows. Females showed a greater number of long COVID-19 symptoms, assumed more psychotropic medications, and showed a greater severity of psychiatric symptoms than males. In the hospitalized subjects, females showed stronger correlations between DH and the number of medications taken and psychotropic medications taken during long COVID-19 than males. Only the females showed a negative relationship between DH and the severity of agitated depression during long COVID-19, whereas no significant relationship was present in the males.

The present findings corroborate the higher prevalence of long COVID-19 symptoms, and specifically psychiatric symptoms, in females compared with males. Sylvester et al. [26] found that COVID-19 sequelae in the psychiatric or mood, musculoskeletal, and respiratory categories were more prevalent in females compared with males. Moreover, females generally showed a higher probability of developing long COVID-19 [26]. A prospective cohort study by Bai et al. found that females have a threefold higher risk of developing long COVID compared with males [27]. In both studies, anxiety and depression were the most frequent psychiatric symptoms. Mazza et al. [28] found that females and patients with positive previous psychiatric diagnoses were more likely to develop psychiatric symptoms during long COVID-19.

Gender differences in immune system response might account for the results found [24,25,26]. Females embed a more innate and adaptive immune response, which contributes to their increased susceptibility to inflammatory diseases [27]. Consequently, it might cause a greater vulnerability to disorders in which the immune system is chronically activated, such as in long COVID-19 [29,30,31]. In fact, the emergence of psychiatric symptoms has been hypothesized to derive from a strong immune response to SARS-CoV-2, which may cause chronic inflammatory processes and neurodegeneration [32]. Moreover, as certain symptoms related to long COVID-19 might resemble those of perimenopause and menopause [33], sex hormone differences might also play a role in the results found. Specifically, a temporary disruption to physiological ovarian steroid hormone production can be hypothesized [34], as decreased levels of estrogens during COVID-19 infection may reflect a greater sensibility to inflammatory diseases and their consequences, as described above [31]. Many studies [35,36] suggest the potential role of estrogen in the treatment of COVID-19 and its sequelae. Therefore, a decrease in inflammatory levels might lead to a decrease in the symptom burden associated with long COVID-19.

Gender-specific differences in the microbiome have also been shown to play a role in both the development of long COVID-19 and depression [37,38]. Niemela et al. [37] found some gender-specific alterations in the microbiome to be associated with the development of depression. COVID-19 has been shown to be associated with microbiome alterations and gut barrier dysfunction in human studies [38], which could increase the translocation of bacterial products and toxins into the circulatory system and exacerbate the systemic inflammatory response. Specifically, Zhang et al. [38] found that fatigue and neuropsychiatric symptoms in long COVID-19 were associated with increased levels of nosocomial pathogens such as *Clostridium innocuum* and *Actinomyces naeslundii.* Further studies are needed to investigate the relationship among the microbiome, depression, and COVID-19, as well as the potential diagnostic and therapeutic implications.

Lastly, some sociocultural factors should also be taken into consideration. According to Gebhard et al. [39], there are some specific gender-related sociocultural parameters which predict a higher risk of developing long COVID-19 in women. In particular, living alone, higher stress levels, and lower education predicted higher probability of developing long COVID-19 symptoms. Hence, some psychosocial gender differences might play a role in developing anxiety and depressive symptoms after COVID-19.

Different from what was expected, no main effect from DH was observed. The results found were in line with those of Huarcaya-Victoria et al. [7] and in contrast to those of Fernández-de-Las-Peñas et al. [15]. Differences with the latter might be explained by the different tools used to assess the severity of psychiatric symptoms. Firstly, Fernández-de-Las-Peñas et al. [15] used self-administered scales collected through telephone interviews, whereas in the present study, the scales used were clinician-based and included patient observation. Self-administered scales capture the subject’s internal feelings, which may not match the physician’s observations. DH might relate more to such internal feelings rather than to the signs and symptoms captured by clinician-administered rating scales. Additional studies combining self-reporting and clinician-based rating scales are necessary to clarify the role of DH. Additionally, the present study did not consider the severity parameters related to DH. Even though a long DH is considered a parameter of severity in COVID-19 inpatients, other factors, such as a patient’s age and availability of resources [40], might modulate its impact on inpatients. DH might be also modulated by other factors, such as disease burden during a hospital stay, admission to an intensive care unit (ICU), alterations in gas exchange, and laboratory parameters. All of these factors have been proven to influence long COVID-19’s onset and course [7,41,42], and therefore, further studies including these parameters along with DH might be helpful in clarifying the impact of such variables on symptoms persisting after COVID-19.

Non-hospitalized females showed higher levels of agitated depression during long COVID-19 compared with non-hospitalized males. Agitated depression in COVID-19 patients has been poorly investigated, but it seems to be more prevalent when compared with the general population [43]. Agitated depression, also known as mixed depression [19,44], is a sub-form of depression mixing the core features of depression, such as low mood, anhedonia, and lack of motivation, with excitatory symptoms (i.e., psychic and motor agitation, inner tension, early insomnia, and lack of retardation) [19,41]. Agitated depressive symptoms are associated with greater levels of functional impairment, lower quality of life, and greater risk of suicide [45,46,47,48,49,50], and in the context of long COVID-19, they are associated with a greater disease burden [51]. The neurobiology underlying agitated depression involves a large array of biological alterations, including monoaminergic hyperactivation, circadian rhythm disruption, and hormonal and immune system dysregulation [44]. Specifically, greater levels of proinflammatory cytokines (i.e., tumor necrosis factor- (TNF-) and interleukin-18 (IL-18)) have been shown to relate to the severity of agitated depression [6]. TNF- and IL-18 are two pivotal components of the cytokine storm associated with SARS-CoV-2 infection [52,53]. High levels of these cytokines have been shown in subjects hospitalized for COVID-19 [52,53], as well as during long COVID-19 [54]. During hospitalization, the levels of TNF- have been shown to be fourfold higher in females than males [55], possibly because of the effect of sex hormones. Testosterone has been shown to exert a buffering effect on the TNF- increase [56]. The level of TNF-α is higher in adult men with lower testosterone levels [57], while the expression of TNF-α is inhibited by testosterone in men with hypogonadism [58]. Conversely, female hormones such as 17-estradiol have been shown to increase the levels of TNF-α [59]. Therefore, the higher severity of agitated depression in females could be driven by gender-specific high levels of TNF-α during COVID-19, which could remain high after SARS-CoV2 infection. Gender differences regarding the severity of agitated depression are not present in subjects with a history of hospital admission for COVID-19.

On the other hand, hospitalized females showed an inverse relationship between the severity of agitated depression and DH, whereas no relationship was found in males. Mitrović-Ajtić et al. [55] and Alonso-Domínguez et al. [54] showed a progressive reduction in TNF-α in hospitalized subjects along with estradiol levels [60]. Treatments aimed at reducing COVID-19 symptoms in hospitalized patients, such as corticosteroids or specific TNF-α antagonists like infliximab, have been shown to reduce TNF-α [61,62]. Therefore, prolonged hospitalization may lead to prolonged exposure to treatments aimed at decreasing cytokine storms. This will lower the levels of the proinflammatory cytokines involved in the development of agitated depressive symptoms once the infection is extinguished. Additionally, DH in females was also related to a greater number of psychotropic drugs prescribed during and after having COVID-19. Hence, a correct psychopharmacological treatment could be responsible for the low levels of mixed symptoms in those patients. Lastly, agitated depression is characterized by a high level of energy [63]. Therefore, the physical burden derived from long hospitalization for COVID-19 and long COVID-19 symptoms may significantly reduce the energy levels in those patients, who might experience forms of melancholic or anxious depression more frequently.

Some limitations should be laid out. First, the cross-sectional design impedes an exhaustive clarification of the relationship under investigation in the present work. Additionally, the lack of a control group and the potential selection bias might reduce the internal validity and generalizability of the study. Third, as mentioned above, the study’s variables did not include factors that might influence long COVID-19-related psychopathology, such as symptom severity, laboratory testing, and respiratory parameters. Moreover, it was not possible to provide the levels of blood inflammatory cytokines such as TNF-α. Therefore, the explanation of possible mechanisms related to the combined effect of DH and gender on the severity of agitated depression has to be considered speculative. On the other hand, the strengths of this study rely on the large sample size and the thorough clinical evaluation including several specialists. Furthermore, the scales chosen gave a comprehensive description of the psychiatric symptoms’ severity, thus giving a detailed and measurable parameter for psychiatric symptom severity.

## 5. Conclusions

This study’s findings corroborate the existing evidence of a gender effect on the severity of long COVID-19 psychiatric symptoms. Furthermore, the interplay between gender and DH modulates the severity of agitated depression, whereas DH alone does not affect long COVID-19-associated psychopathology. These findings have several implications. First, greater attention should be given to women admitted to hospitals for SARS-CoV-2. These subjects will be more at risk of developing severe forms of long COVID-19, and therefore, close monitoring will be needed to ensure prompt intervention in the case of development of this syndrome. Second, the present findings might foster research for possible common mechanisms connecting COVID-19-related cytokine storms, sex hormones, and psychiatric symptoms. This would help with finding treatments aimed to reduce COVID-19’s illness burden and its sequelae. Third, the relationship found between DH, gender, and agitated depression gives more insight into the pathophysiology of this severe and underestimated specific form of depression. Agitated depression is a severe form related to greater risk of suicide [2,64]. Suicidal behaviors are also higher in females than males [63]. The present study demonstrated that prolonged exposure to treatments for COVID-19 might reduce the severity of agitated depressive symptoms in women. Such findings suggest that a greater length of treatment for women hospitalized for COVID-19 might be given to confer resilience against the onset and progression of agitated depression in this population. This will turn in lower the risk of suicidal thoughts days or months after contracting SARS-CoV-2. Further research is needed to confirm the aforementioned relationship and to understand its possible shared pathophysiological mechanisms. 

## Figures and Tables

**Table 1:** Differences in sociodemographic and clinical variables related to COVID-19 and long COVID-19 among HM, NHM, HF and NHF.

	NHM (n = 218)	HM (n = 255)	NHF (n = 278)	HF (n = 245)	*F*	*p*-Value	Post-Hoc											
							nHM vs. HM		NHM vs. NHF		NHM vs. HF		HM vs. NHF		HM vs. HF		NHF vs. HF	
							*p*	d	*p*	d	*p*	d	*p*	d	*p*	d	*p*	d
Age, mean ± SD	51.70 ± 10.41	51.06 ± 8.08	53.44 ± 8.95	53.55 ± 10.31	4.436	**0.004**	0.883	0.07	0.177	0.17	0.153	0.18	**0.019**	**0.28**	**0.017**	**0.267**	0.999	0.011
Education (yrs), mean ± SD	13.89 ± 5.44	13.49 ± 4.5	14.04 ± 4.84	13.79 ± 4.40	0.620	0.602	0.799	0.08	0.986	0.03	0.996	0.02	0.544	0.118	0.896	0.067	0.933	0.054
Occupation, n (%)	182 (83.49)	226 (88.63)	222 (79.86)	168 (68.57)	33.752	**<0.001**	0.11	0.07	0.30	0.45	**<0.001**	**0.47**	**0.006**	**0.429**	**<0.001**	**0.457**	**0.003**	**0.19**
Psychiatric history prior to COVID, n (%)	14 (6.4)	17 (6.7)	33 (11.9)	31 (12.7)	9.387	**0.025**	0.91	0.03	**0.04**	0.17	**0.02**	**0.19**	**0.04**	**0.165**	**0.02**	**0.11**	0.67	0.05
Psychotropic drugs prior to COVID, n (%)	6 (2.75)	11 (4.3)	15 (5.39)	19 (7.76)	6.377	0.095	0.36	**0.04**	0.15	0.12	0.58	0.16	0.54	0.105	0.64	0.08	0.28	0.13
Substance abuse n (%)	34 (15.6)	61 (23.92)	30 (10.79)	29 (11.84)	20.930	**<0.001**	**0.02**	0.25	0.11	0.19	0.238	0.20	**<0.001**	**0.25**	**<0.001**	**0.15**	0.704	0.16
Days from COVID onset, mean ± SD	152.78 ± 112.9	138.36 ± 95.55	157.78 ± 113.37	145.88 ± 97.55	1.651	0.176	0.446	0.14	0.958	0.004	0.895	0.07	0.151	0.185	0.855	0.078	0.582	0.113
Symptoms during COVID, mean ± SD	7.77 ± 4.184	8.01 ± 3.762	10.07 ± 4.225	9.77 ± 4.147	20.890	**<0.001**	0.922	0.06	**<0.001**	0.06	**<0.001**	**0.04**	**<0.001**	**0.515**	**<0.001**	**0.445**	0.835	0.07
Psychiatric symptoms during COVID, n (%)	5 (2.29)	9 (3.53)	2 (0.004)	12 (4.9)	9.052	**0.029**	0.43	0.09	0.14	0.09	0.14	0.12	**0.02**	**0.12**	0.45	**0.09**	**0.003**	**0.12**
Psychotropic drugs during COVID, n (%)	9 (4.12)	11 (4.31)	25 (8.99)	20 (8.16)	7.903	**0.048**	0.92	0.11	**0.03**	0.10	**0.07**	**0.16**	**0.06**	**0.08**	**0.08**	**0.10**	0.73	0.10
Post-COVID symptoms, mean ± SD	3.02 ± 2.91	3.03 ± 2.66	4.98 ± 3.59	4.6 ± 3.48	26.06	**<0.001**	1.000	0.01	**<0.001**	0.06	**<0.001**	**0.48**	**<0.001**	**0.617**	**<0.001**	**0.507**	0.514	0.098
Post-COVID drugs, mean ± SD	1.53 ± 2.14	1.75 ± 2.35	1.71 ± 2.01	2.29 ± 2.73	4.723	0.003 *	0.723	0.09	0.832	0.09	0.003	0.31	0.996	0.212	0.051	0.212	0.024	0.244
Number of post-COVID psychotropic drugs, mean ± SD	0.05 ± 0.231	0.07 ± 0.331	0.18 ± 0.579	0.18 ± 0.513	6.372	**<0.001**	0.958	0.07	**0.005**	0.29	**0.007**	**0.33**	**0.018**	**0.233**	**0.024**	**0.212**	1.000	-
BPRS, mean ± SD	25.90 ± 3.73	26.15 ± 3.45	27.78 ± 4.41	27.32 ± 4.54	12.347	**<0.001**	0.909	0.07	**<0.001**	0.07	**0.001**	**0.34**	**<0.001**	**0.412**	**0.007**	**0.29**	0.580	0.103
HAM-D, mean ± SD	4.78 ± 3.71	5.13 ± 4.02	6.66 ± 4.57	6.95 ± 4.43	10.860	**<0.001**	0.903	0.09	**<0.001**	0.45	**<0.001**	**0.53**	**0.002**	**0.36**	**<0.001**	**0.43**	0.891	0.064
HAM-A, mean ± SD	5.50 ± 5.09	5.77 ± 4.84	8.16 ± 5.96	8.15 ± 5.81	13.180	**<0.001**	0.970	0.05	**<0.001**	0.48	**<0.001**	**0.48**	**<0.001**	**0.44**	**<0.001**	**0.445**	1.000	0.002
KMDRS, mean ± SD	4.41 ± 2.20	4.94 ± 2.16	5.27 ± 3.24	4.87 ± 2.63	4.436	**<0.001**	0.123	0.24	**<0.001**	0.31	0.226	0.19	0.228	0.119	0.992	0.029	0.308	0.136

Legend: BPRS, Brief-Psychiatric Rating Scale; HAM-A, Hamilton Anxiety Rating Scale; HAM-D, Hamilton Rating Scale For Depression; KMDRS, Koukopoulos Mixed Depression Rating Scale; HM, Hospitalized Male; NHM, Non Hospitalized Male; HF, Hospitalized Female; NHF, Non Hospitalized Female; SD, Standard Deviation; yrs, years. * After adjusting for potential confounding variables, data loses statistical significance.

**Table 2:** Multivariate regression analyses–Relationship between gender, DH and gender-DH interaction with variables related to Long COVID outcomes.

DV	Predictor	B	SE	t	*p*	95% CI
Number of long COVID-19 symptoms	Gender	1.703	0.326	5.231	**<0.001**	1.0642; 0.343
	DH	0.006	0.015	0.410	0.682	−0.024; 0.037
	Gender × DH interaction	−0.008	0.009	−0.860	0.390	−0.026; 0.010
Number of medications during long COVID-19	Gender	0.192	0.257	0.746	0.456	−0.313; 0.696
	DH	0.008	0.012	−0.683	0.495	−0.032; 0.015
	Gender × DH interaction	0.02	0.007	2.799	**0.005**	0.006; 0.035
Number of psychotropic medications during long COVID-19	Gender	0.091	0.045	2.039	**0.042**	0.003; 0.179
	DH	0.000	0.002	0.185	0.853	−0.004; 0.005
	Gender × DH interaction	0.001	0.001	1.093	0.275	−0.001; 0.004
BPRS	Gender	1.339	0.425	3.150	**0.002**	0.504; 2.174
	DH	0.018	0.02	0.897	0.37	−0.022; 0.058
	Gender × DH interaction	−0.009	0.012	−0.704	0.482	−0.032; 0.015
HAM-A	Gender	2.635	0.630	4.184	**<0.001**	1.397; 3.873
	DH	0.019	0.028	0.671	0.503	−0.036; 0.073
	Gender × DH interaction	−0.014	0.017	−0.835	0.404	−0.047; 0.019
HAM-D	Gender	1.698	0.535	3.172	**0.002**	0.645; 2.750
	DH	0.021	0.012	1.751	0.081	−0.003; 0.044
	Gender × DH interaction	0.010	0.017	0.614	0.540	−0.023; 0.043
KMDRS	Gender	0.242	0.252	0.961	0.337	−0.235; 0.736
	DH	0.021	0.012	1.751	0.081	−0.003; 0.044
	Gender × DH interaction	-0.017	0.007	−2.395	**0.017**	−0.031; −0.003

Legend: significant results are in bold. BPRS, Brief-Psychiatric Rating Scale; HAM-A, Hamilton Anxiety Rating Scale; HAM-D, Hamilton Rating Scale for Depression; KMDRS, Koukopoulos Mixed Depression Rating Scale; DH, Duration of Hospitalization.

**Table 3:** Regression between DH and variables related to long COVID-19 outcomes in males and females.

**Males**					
**Dependent variable**	**B**	**SE**	**β**	***p***	**95% CI**
Number of medications during long COVID-19	0.012	0.005	0.144	**0.023**	0.002; 0.023
Number of psychotropic medications during long COVID-19	0.002	0.001	0.147	**0.019**	0.000; 0.003
KMDRS	0.004	0.005	0.046	0.466	−0.006; 0.013
**Females**					
**Dependent variable**	**B**	**SE**	**β**	***p***	**95% CI**
Number of medications during long COVID-19	0.033	0.005	0.394	**<0.001**	0.023; 0.043
Number of psychotropic medications during long COVID-19	0.003	0.001	0.203	**0.001**	0.001; 0.005
KMDRS	−0.014	0.005	−0.169	**0.008**	−0.023; −0.004

Legend: significant results are in bold. KMDRS: Koukopoulos Mixed Depression Rating Scale.

## Data Availability

The original contributions presented in this study are included in the article. Further inquiries can be directed to the corresponding author.

## Supplementary Materials

The following supporting information can be downloaded at https://www.mdpi.com/article/10.3390/brainsci14080744/s1. Table S1. Analyses of covariance–Corrections of results for possible confounding variables. Table S2. Linear regressions between age and variables related to long COVID-19. Table S3. Linear regressions between gender and variables related to long COVID-19. Table S4. Linear regressions between education years and variables related to long COVID-19. Table S5. Linear regressions between occupation and variables related to long COVID-19. Table S6. Linear regressions between psychiatric history prior to COVID-19 and variables related to long COVID-19. Table S7. Linear regressions between psychotropic medications assumption prior to COVID-19 and variables related to long COVID-19. Table S8. Linear regressions between substance abuse before COVID-19 and variables related to long COVID-19. Table S9. Linear regressions between distance from COVID-19 onset and variables related to long COVID-19. Table S10. Linear regressions between history of psychiatric symptoms during COVID-19 and variables related to long COVID-19. Table S11. Linear regressions between number of symptoms during COVID-19 and variables related to long COVID-19. Table S12. Linear regressions between psychiatric medications during COVID-19 and variables related to long COVID-19. Table S13. Analysis of linear regressions between the duration of hospitalization and Long COVID outcomes in males. Table S14. Analysis of linear regressions between the duration of hospitalization and Long COVID outcomes in females. Table S15. Multivariate regression analyses–Results adjusted for variables showing a significant relationship with variables related to long COVID-19 in linear regressions. Table S16. Multivariate regression analyses–Results adjusted for variables showing a significant relationship with each Long COVID outcomes in the linear regressions. Table S17. Multivariate regression analyses–Results adjusted for variables showing a significant relationship with each Long COVID outcomes in the linear regressions. Table S18. Multivariate regression analyses–Results adjusted for variables showing a significant relationship with each Long COVID outcomes in the linear regressions. Table S19. Multivariate regression analyses–Results adjusted for variables showing a significant relationship with each Long COVID outcomes in the linear regressions. Table S20. Multivariate regression analyses–Results adjusted for variables showing a significant relationship with each Long COVID outcomes in the linear regressions. Table S21. Multivariate regression analyses–Results adjusted for variables showing a significant relationship with each Long COVID outcomes in the linear regressions.
