# Supplementary material for: The Interplay between Gender and Duration of Hospitalization Modulates Psychiatric Symptom Severity in Subjects with Long COVID-19
Source: Brain Sci. 2024 Jul 25;14(8):744. doi: 10.3390/brainsci14080744 (PMC11352493; doi:10.3390/brainsci14080744)
Supplement: Supplementary file 1 [file brainsci-14-00744-s001.zip › brainsci-3088729-supplementary.pdf]

Supplement

Table S1. **Analyses of covariance – Corrections of results for possible confounding variables.**

	NHM (n=218)	HM (n=255)	NHF (n=278)	HF (n=245)	F	p-value
**Number of long COVID-19 symptoms, mean ± SD**	3.02 ± 2.91	3.03 ± 2.66	4.98 ± 3.59	4.6 ± 3.48	9.115	**<0.001**
**Number of medications during long COVID-19, mean ± SD**	1.53±2.14	1.75±2.35	1.71±2.01	2.29±2.73	2.414	0.065
**Number of psychotropic medications during long COVID-19, mean ± SD**	0.05±0.231	0.07±0.331	0.18±0.579	0.18±0.513	2.798	**0.039**
**BPRS, mean ± SD**	25.90±3.73	26.15±3.45	27.78±4.41	27.32±4.54	4.164	**0.006**
**HAM-D, mean ± SD**	4.78±3.71	5.13±4.02	6.66±4.57	6.95±4.43	3.730	**0.011**
**HAM-A, mean ± SD**	5.50±5.09	5.77±4.84	8.16±5.96	8.15±5.81	5.208	**0.001**
**KMDRS, mean ± SD**	4.41±2.20	4.94±2.16	5.27±3.24	4.87±2.63	2.985	**0.030**

Legend: significant results are in bold. BPRS, Brief-Psychiatric Rating Scale; HAM-A, Hamilton Anxiety Rating Scale; HAM-D, Hamilton Rating Scale for Depression; KMDRS, Koukopoulos Mixed Depression Rating Scale.

Table S2: Linear regressions between age and variables related to long COVID-19

**Dependent variable**	**B**	**SE**	**β**	**P**	**95% CI**
**Number of long COVID-19 symptoms**	-0.013	0.015	-0.038	0.392	-0.043<br>0.017
**Number of medications during long COVID-19**	0.066	0.012	0.240	**0.000**	0.042<br>0.089
**Number of psychotropic medications during long COVID-19**	0.067<br>0.003	0.002	0.067	0.138	0.000<br>0.007
**BPRS**	0.000	0.020	-0.002	0.971	-0.039<br>0.038
**HAM-A**	-0.044	0.030	-0.075	0.138	-0.103<br>0.014
**HAM-D**	-0.018	0.025	-0.038	0.473	-0.066<br>0.031
**KMDRS**	-0.009	0.012	-0.033	0.455	-0.031<br>0.014

Legend: significant results are in bold. BPRS, Brief-Psychiatric Rating Scale; HAM-A, Hamilton Anxiety Rating Scale; HAM-D, Hamilton Rating Scale for Depression; KMDRS, Koukopoulos Mixed Depression Rating Scale.

Table S3. Linear regressions between gender and variables related to long COVID-19					
**Dependent variable**	**B**	**SE**	**β**	**P**	**95% CI**
**Number of long COVID-19 symptoms**	1.565	0.276	0.246	**0.000**	1.021<br>2.108
**Number of medications during long COVID-19**	0.536	0.229	0.105	0.200	0.086<br>0.985
**Number of psychotropic medications during long COVID-19**	0.113	0.038	0.131	**0.003**	0.037<br>0.188
**BPRS**	1.174	0.360	0.145	**0.001**	0.466<br>1.882
**HAM-A**	2.370	0.541	0.215	**0.000**	1.306<br>3.433
**HAM-D**	1.825	0.447	0.211	**0.000**	0.946<br>2.704
**KMDRS**	-0.068	0.215	-0.014	0.753	-0.490<br>0.355

Legend: significant results are in bold. BPRS, Brief-Psychiatric Rating Scale; HAM-A, Hamilton Anxiety Rating Scale; HAM-D, Hamilton Rating Scale for Depression; KMDRS, Koukopoulos Mixed Depression Rating Scale.

Table S4. Linear regressions between education years and variables related to long COVID-19					
**Depndent Variable**	**B**	**SE**	**β**	**P**	**95% CI**
**Number of long COVID-19 symptoms**	0.110	0.320	0.160	0.728	-0.520<br>0.740
**Number of medications during long COVID-19**	-0.032	0.026	-0.056	0.220	-0.082<br>0.019
**Number of psychotropic medications during long COVID-19**	0.001	0.004	0.006	0.888	-0.008<br>0.009
**BPRS**	-0.095	0.041	-0.105	**0.020**	-0.175<br>-0.015
**HAM-A**	-0.057	0.062	-0.046	0.358	-0.179<br>0.065
**HAM-D**	-0.035	0.051	-0.036	0.494	-0.136<br>0.066
**KMDRS**	-0.045	0.024	-0.083	0.063	-0.092<br>0.002

Legend: significant results are in bold. BPRS, Brief-Psychiatric Rating Scale; HAM-A, Hamilton Anxiety Rating Scale; HAM-D, Hamilton Rating Scale for Depression; KMDRS, Koukopoulos Mixed Depression Rating Scale.

Table S5. Linear regressions between occupation and variables related to long COVID-19					
**Dependent variable**	**B**	**SE**	**β**	**P**	**95% CI**
**Number of long COVID-19 symptoms**	-0.101	0.349	-0.013	0.773	-0.786<br>0.585
**Number of medications during long COVID-19**	-0.957	0.278	-0.154	**0.001**	-10.503<br>-0.412
**Number of psychotropic medications during long COVID-19**	-0.118	0.030	-0.172	**0.000**	-0.178<br>-0.059
**BPRS**	-10.730	0.439	-0.174	**0.000**	-20.592<br>-0.868
**HAM-A**	-10.034	0.676	-0.077	0.127	-20.362<br>0.295
**HAM-D**	-10.549	0.553	-0.146	**0.005**	-20.637<br>-0.461
**KMDRS**	-0.129	0.263	-0.022	0.625	-0.645<br>0.388

Legend: significant results are in bold. BPRS, Brief-Psychiatric Rating Scale; HAM-A, Hamilton Anxiety Rating Scale; HAM-D, Hamilton Rating Scale for Depression; KMDRS, Koukopoulos Mixed Depression Rating Scale.

Table S6. Linear regressions between psychiatric history prior to COVID-19 and variables related to long COVID-19					
**Dependent Variable**	**B**	**SE**	**β**	**P**	**95% CI**
**Number of long COVID-19 symptoms**	0.328	0.484	0.030	0.498	-0.623<br>10.278
**Number of medications during long COVID-19**	-0.087	0.390	-0.010	0.824	-0.852<br>0.679
**Number of psychotropic medications during long COVID-19**	0.095	0.066	0.065	0.148	-0.034<br>0.224
**BPRS**	10.524	0.614	0.111	**0.013**	0.317<br>20.731
**HAM-A**	50.125	0.904	0.275	**0.000**	30.347<br>60.903
**HAM-D**	20.781	0.762	0.189	**0.000**	10.282<br>40.280
**KMDRS**	0.351	0.365	0.043	0.366	-0.365<br>10.067

Legend: significant results are in bold. BPRS, Brief-Psychiatric Rating Scale; HAM-A, Hamilton Anxiety Rating Scale; HAM-D, Hamilton Rating Scale for Depression; KMDRS, Koukopoulos Mixed Depression Rating Scale.

Table S7. Linear regressions between psychotropic medications assumption prior to COVID-19 and variables related to long COVID-19

Dependent variable	B	SE	β	P	95% CI
**Number of long COVID-19 symptoms**	0.511	0.600	0.038	0.395	-0.668<br>10.689
**Number of medications during long COVID-19**	-0.089	0.483	-0.008	0.854	-10.038<br>0.861
**Number of psychotropic medications during long COVID-19**	0.224	0.018	0.123	**0.006**	0.065<br>0.384
**BPRS**	20.065	0.761	0.121	**0.007**	0.570<br>30.560
**HAM-A**	40.989	10.139	0.216	**0.000**	20.749<br>70.229
**HAM-D**	20.968	0.950	0.163	**0.002**	10.100<br>40.837
**KMDRS**	0.838	0.451	0.083	0.064	-0.048<br>10.724

Legend: significant results are in bold. BPRS, Brief-Psychiatric Rating Scale; HAM-A, Hamilton Anxiety Rating Scale; HAM-D, Hamilton Rating Scale for Depression; KMDRS, Koukopoulos Mixed Depression Rating Scale.

Table S8. Linear regressions between substance abuse before COVID-19 and variables related to long COVID-19 .

Dependent variable	B	SE	β	P	95% CI
**Number of long COVID-19 symptoms**	-0.851	0.369	-0.103	**0.022**	-10.576<br>-0.126
**Number of medications during long COVID-19**	-10.037	0.295	-0.157	**0.000**	-10.617<br>-0.457
**Number of psychotropic medications during long COVID-19**	-0.095	0.050	-0.084	0.061	-0.193<br>0.004
**BPRS**	-0.791	0.473	-0.075	0.095	-10.720<br>0.137
**HAM-A**	0.032	0.721	0.002	0.965	-10.386<br>10.449
**HAM-D**	-0.368	0.595	-0.033	0.536	-10.537<br>0.801
**KMDRS**	0.058	0.280	0.009	0.836	-0.492<br>0.608

Legend: significant results are in bold. BPRS, Brief-Psychiatric Rating Scale; HAM-A, Hamilton Anxiety Rating Scale; HAM-D, Hamilton Rating Scale for Depression; KMDRS, Koukopoulos Mixed Depression Rating Scale.

Table S9. Linear regressions between distance from COVID-19 onset and variables related to long COVID-19					
**Dependent variable**	**B**	**SE**	**β**	**P**	**95% CI**
**Number of long COVID-19 symptoms**	0.005	0.001	0.158	**0.000**	0.002<br>0.008
**Number of medications during long COVID-19**	0.002	0.001	0.074	0.104	0.000<br>0.004
**Number of psychotropic medications during long COVID-19**	0.001	0.000	0.153	**0.001**	0.000<br>0.001
**BPRS**	0.001	0.002	0.015	0.739	-0.003<br>0.004
**HAM-A**	-0.008	0.003	-0.132	**0.008**	-0.013<br>-0.002
**HAM-D**	-0.004	0.002	-0.088	0.096	-0.009<br>0.001
**KMDRS**	0.000	0.001	-0.028	0.526	-0.003<br>0.001

Legend: significant results are in bold. BPRS, Brief-Psychiatric Rating Scale; HAM-A, Hamilton Anxiety Rating Scale; HAM-D, Hamilton Rating Scale for Depression; KMDRS, Koukopoulos Mixed Depression Rating Scale.

Table S10 Linear regressions between history of psychiatric symptoms during COVID-19 and variables related to long COVID-19					
**Dependent variable**	**B**	**SE**	**β**	**P**	**95% CI**
**Number of long COVID-19 symptoms**	20.249	0.704	0.142	**0.001**	0.867<br>30.631
**Number of medications during long COVID-19**	0.789	0.572	0.062	0.168	-0.335<br>10.912
**Number of psychotropic medications during long COVID-19**	0.320	0.096	0.148	**0.001**	0.132<br>0.508
**BPRS**	0.437	0.908	0.022	0.630	-10.346<br>20.220
**HAM-A**	0.011	10.381	0.000	0.994	-20.704<br>20.726
**HAM-D**	10.256	10.138	0.058	0.270	-0.981<br>30.493
**KMDRS**	-0.252	0.536	-0.021	0.638	-0.304<br>0.800

Legend: significant results are in bold. BPRS, Brief-Psychiatric Rating Scale; HAM-A, Hamilton Anxiety Rating Scale; HAM-D, Hamilton Rating Scale for Depression; KMDRS, Koukopoulos Mixed Depression Rating Scale.

Table S11. Linear regressions between number of symptoms during COVID-19 and variables related to long COVID-19

Dipendent variable	B	SE	β	P	95% CI
**Number of long COVID-19 symptoms**	0.390	0.031	0.496	**0.000**	0.330<br>0.450
**Number of medications during long COVID-19**	0.013	0.028	0.021	0.644	-0.043<br>0.069
**Number of psychotropic medications during long COVID-19**	0.005	0.003	0.073	0.103	-0.001<br>0.011
**BPRS**	0.136	0.045	0.136	**0.002**	0.049<br>0.224
**HAM-A**	0.288	0.067	0.212	**0.000**	0.157<br>0.420
**HAM-D**	0.228	0.055	0.213	**0.000**	0199<br>0.336
**KMDRS**	0.068	0.026	0.115	**0.010**	0.017<br>0.120

Legend: significant results are in bold. BPRS, Brief-Psychiatric Rating Scale; HAM-A, Hamilton Anxiety Rating Scale; HAM-D, Hamilton Rating Scale for Depression; KMDRS, Koukopoulos Mixed Depression Rating Scale.

Table S12. Linear regressions between psychiatric medications during COVID-19 and variables related to long COVID-19

Dependent variable	B	SE	β	P	95% CI
**Number of long COVID-19 symptoms**	10.451	0.588	0.110	**0.014**	0.296<br>20.605
**Number of medications during long COVID-19**	20.078	0.467	0.198	**0.000**	10.160<br>20.996
**Number of psychotropic medications during long COVID-19**	10.028	0.066	0.573	**0.000**	0.898<br>10.157
**BPRS**	10.851	0.750	0.110	**0.014**	0.377<br>30.326
**HAM-A**	10.046	10.148	0.046	0.363	-10.211<br>30.303
**HAM-D**	20.86310	0.942	0.112	20.133	0.157<br>30.6
**KMDRS**	-0.078	0.446	-0.008	0.861	-0.954<br>0.798

Legend: significant results are in bold. BPRS, Brief-Psychiatric Rating Scale; HAM-A, Hamilton Anxiety Rating Scale; HAM-D, Hamilton Rating Scale for Depression; KMDRS, Koukopoulos Mixed Depression Rating Scale.

**Table S13.** Analysis of linear regressions between the duration of hospitalization and Long COVID outcomes in males.					
**Dependent variable**	**B**	**SE**	**β**	**P**	**95% CI**
**Number of drugs post-COVID**	0.012	0.005	0.144	**0.023**	0.002<br>0.023
**Number of psychotropic drugs post-COVID**	0.002	0.001	0.147	**0.019**	0.000<br>0.003
**KMDRS**	0.004	0.005	0.046	0.466	-0.006<br>0.013
**Legend:** significant results are in bold. KMDRS, Koukopoulos Mixed Depression Rating Scale.					

**Table S14. Analysis of linear regressions between the duration of hospitalization and Long COVID outcomes in females.**					
**Dependent variable**	**B**	**SE**	**β**	**P**	**95% CI**
**Number of drugs post-COVID**	0.033	0.005	0.394	**0.000**	0.023<br>0.043
**Number of psychotropic drugs post-COVID**	0.003	0.001	0.203	**0.001**	0.001<br>0.005
**KMDRS**	-0.014	0.005	-0.169	**0.008**	-0.024<br>-0.004
**Legend:** significant results are in bold. KMDRS, Koukopoulos Mixed Depression Rating Scale.					

**Table S15 – Multivariate regression analyses – Results adjusted for variables showing a significant relationship with variables related to long COVID-19 in linear regressions.**					
**Number of medications during long COVID-19**	B	SE	t	P	95% CI
**Age**	0.053	0.011	4.617	**0.000**	0.030<br>0.075
**Occupation**	-0.523	0.267	-1.955	0.051	-1.048<br>0.003
**Substance abuse prior to COVID-19**	-0.571	0.277	-2.063	**0.040**	-1.114<br>-0.027
**Number of psychotropic medications during COVID-19**	1.617	0.434	3.727	**0.000**	0.764<br>2.470
**Gender**	-0.158	0.253	-0.626	0.532	-0.655<br>0.399
**DH**	-0.009	0.012	-0.806	0.421	-0.032<br>0.013
**Gender*DH**	0.020	0.007	2.0845	**0.005**	0.006<br>0.034
**Legend:** significant results are in bold. BPRS, Brief-Psychiatric Rating Scale; HAM-A, Hamilton Anxiety Rating Scale; HAM-D, Hamilton Rating Scale for Depression; KMDRS, Koukopoulos Mixed Depression Rating Scale; DH, Duration of Hospitalization					

**Table S16 – Multivariate regression analyses – Results adjusted for variables showing a significant relationship with each Long COVID outcomes in the linear regressions.**					
**Dependent variable: Number of psychotropic medications during long COVID-19**	B	SE	t	P	95% CI
**Psychotropic medications prior to COVID-19**	0.199	0.064	3.113	**0.002**	0.073<br>0.325
**Distance from COVID-19 onset**	0.000	0.000	2.211	**0.027**	0.00003930<br>0.001
**Psychotropic medications during COVID-19**	0.963	0.064	15.170	**0.000**	0.839<br>1.088
**Gender**	0.028	0.036	0.790	0.430	-0.042<br>0.099
**DH**	-0.002	0.002	-1.345	0.179	-0.006<br>0.001
**Gender*DH**	0.003	0.001	2.589	**0.010**	0.001<br>0.005
**Legend:** significant results are in bold. BPRS, Brief-Psychiatric Rating Scale; HAM-A, Hamilton Anxiety Rating Scale; HAM-D, Hamilton Rating Scale for Depression; KMDRS, Koukopoulos Mixed Depression Rating Scale; DH, Duration of Hospitalization					

**Table S17 – Multivariate regression analyses – Results adjusted for variables showing a significant relationship with each Long COVID outcomes in the linear regressions.**					
**Dependent variable: KMDRS**	B	SE	t	P	95% CI
**Distance from COVID-19 onset**	0.000	0.001	-0.454	0.650	-0.003<br>0.002
**Psychotropic medications during COVID-19**	-0.056	0.451	-0.123	0.902	-0.941<br>0.830
**number of long COVID-19 symptoms**	0.085	0.035	2.453	**0.015**	0.017<br>0.153
**Gender**	0.098	0.257	0.379	0.705	-0.408<br>0.603
**DH**	0.020	0.012	1.714	0.087	-0.003<br>0.044
**Gender*DH**	-0.017	0.007	-2.311	**0.021**	-0.031<br>-0.002
**Legend:** significant results are in bold. BPRS, Brief-Psychiatric Rating Scale; HAM-A, Hamilton Anxiety Rating Scale; HAM-D, Hamilton Rating Scale for Depression; KMDRS, Koukopoulos Mixed Depression Rating Scale; DH, Duration of Hospitalization					

**Table S18 – Multivariate regression analyses – Results adjusted for variables showing a significant relationship with each Long COVID outcomes in the linear regressions.**					
**Dependent variable: number of long COVID-19 symptoms**	B	SE	t	P	95% CI
**Distance from COVID-19 onset**	0.005	0.001	3.383	**0.001**	0.002<br>0.008
**Psychotropic medications during COVID-19**	0.984	0.575	1.713	0.087	-0.145<br>2.133
**Gender**	1.605	0.323	4.962	**0.000**	0.969<br>2.240
**DH**	0.002	0.015	0.141	0.888	-0.028<br>0.032
**Gender*DH**	-0.007	0.009	-0.736	0.462	-0.025<br>0.011
**Legend:** significant results are in bold. BPRS, Brief-Psychiatric Rating Scale; HAM-A, Hamilton Anxiety Rating Scale; HAM-D, Hamilton Rating Scale for Depression; KMDRS, Koukopoulos Mixed Depression Rating Scale; DH, Duration of Hospitalization					

**Table S19 – Multivariate regression analyses – Results adjusted for variables showing a significant relationship with each Long COVID outcomes in the linear regressions.**					
**Dependent variable: HAM-D**	B	SE	t	P	95% CI
**Distance from COVID-19 onset**	-0.003	0.002	-1.566	0.118	-0.008<br>0.001
**Psychotropic medications prior to COVID-19**	1.049	1.060	0.990	0.323	-1.035<br>3.134
**Gender**	1.599	0.529	3.024	**0.003**	0.599<br>2.638
**DH**	-0.002	0.024	-0.072	0.943	-0.049<br>0.046
**Gender*DH**	0.012	0.017	0.702	0.483	-0.021<br>0.044
**Legend:** significant results are in bold. BPRS, Brief-Psychiatric Rating Scale; HAM-A, Hamilton Anxiety Rating Scale; HAM-D, Hamilton Rating Scale for Depression; KMDRS, Koukopoulos Mixed Depression Rating Scale; DH, Duration of Hospitalization					

**Table S20 – Multivariate regression analyses – Results adjusted for variables showing a significant relationship with each Long COVID outcomes in the linear regressions.**					
**Dependent variable: HAM-A**	B	SE	t	P	95% CI
**Distance from COVID onset**	-0.005	0.003	-2.036	**0.042**	-0.010<br>0.000
**Psychotropic medications prior to COVID-19**	1.201	1.296	0.927	0.355	-1.348<br>3.750
**Gender**	2.424	0.609	3.982	**0.000**	1.227<br>3.621
**DH**	0.020	0.027	0.766	0.444	-0.032<br>0.073
**Gender*DH**	-0.013	0.016	-0.819	0.413	-0.045<br>0.018
**Legend:** significant results are in bold. BPRS, Brief-Psychiatric Rating Scale; HAM-A, Hamilton Anxiety Rating Scale; HAM-D, Hamilton Rating Scale for Depression; KMDRS, Koukopoulos Mixed Depression Rating Scale; DH, Duration of Hospitalization					

Table S21 – Multivariate regression analyses – Results adjusted for variables showing a significant relationship with each Long COVID outcomes in the linear regressions.

Dependent variable: BPRS	B	SE	t	P	95% CI
**Psychotropic medications prior- COVID-19**	1.305	0.958	1.362	0.174	-0.578<br>3.187
**Psychotropic medications during COVID-19**	1.205	0.743	1.621	0.106	-0.256<br>2.665
**number of symptoms During COVID-19**	0.126	0.046	2.753	**0.006**	0.036<br>0.215
**Occupation**	-1.220	0.461	-2.646	**0.008**	-2.125<br>-0.314
**Education**	-0.089	0.041	-2.197	**0.029**	-0.170<br>-0.009
**Psychiatric history prior to COVID-19**	0.606	0.778	0.780	0.436	-0.922<br>2.135
**Gender**	0.770	0.435	1.769	0.077	-0.085<br>1.626
**DH**	0.016	0.020	0.806	0.421	-0.023<br>0.055
**Gender*DH**	-0.007	0.012	-0.575	0.565	-0.030<br>0.017

Legend: significant results are in bold. BPRS, Brief-Psychiatric Rating Scale; HAM-A, Hamilton Anxiety Rating Scale; HAM-D, Hamilton Rating Scale for Depression; KMDRS, Koukopoulos Mixed Depression Rating Scale; DH, Duration of Hospitalization
